# Age-Driven Proteomic Networks in Ningxiang Pig Backfat Identify Candidate Regulators of Carcass Traits

**DOI:** 10.3390/ani16091309

**Published:** 2026-04-24

**Authors:** Lihua Cao, Yu Chen, Qingming Cui, Yuan Deng, Ji Zhu, Huibo Ren, Xionggui Hu, Meizhen Qiu, Xing Zhang, Rongguang Sun, Zhiqiang Tang, Huiming Wang, Yinglin Peng, Chen Chen

**Affiliations:** 1 Hunan Institute of Animal and Veterinary Science, Changsha 410130, China; caolihua@hunaas.cn (L.C.);; 2 Yuelushan Laboratory, Changsha 410128, China; 3 Ningxiang Center for Animal Disease Prevention and Control, Changsha 410600, China; 4 Ningxiang Center for Animal and Aquaculture Affairs, Changsha 410600, China; 5 Hunan Liushahe Ecological Animal Husbandry Co., Ltd., Changsha 410600, China

**Keywords:** adipose tissue, backfat development, carcass traits, lipid metabolism, proteomics

## Abstract

Indigenous pigs constitute crucial genetic reservoirs. Adipose tissue is central to pig growth and metabolism, yet its molecular ontogeny remains poorly characterized in indigenous breeds such as the Ningxiang pig. We employed mass spectrometry to profile backfat proteomes across six postnatal stages (60–360 days). Proteomes clearly separated early (60–120 days) from late stages (300–360 days). Older pigs showed enrichment in processes linked to energy metabolism, translation, immune function, and mitochondrial activity. We identified 43 lipid metabolism proteins exhibiting significant age-dependent abundance. Weighted co-abundance network analysis revealed four protein modules significantly correlated with lean meat percentage, fat percentage, and carcass weight. Intramodular analysis identified four hub proteins—ALDH18A1, FABP4, FBP1, and HADHB—as putative candidates associated with lipid transport, gluconeogenesis, and fatty acid oxidation. This study links temporal proteomic profiles with key carcass traits, providing a data resource and a network-based framework for future research.

## Simple Summary

The backfat of indigenous Ningxiang pigs undergoes substantial molecular remodeling during growth, with energy metabolism and immune function significantly up-regulated as pigs reach maturity. Proteomic analysis identified 43 lipid-related proteins and four hub candidates associated with carcass traits, offering a protein-level framework for studying fat deposition in indigenous breeds.

## 1. Introduction

Adipose tissue, particularly subcutaneous fat, is a metabolically active organ that plays crucial roles in energy storage, endocrine signaling, immune modulation, and thermoregulation [1,2]. In pigs, backfat is not only a major determinant of carcass quality but also an important economic trait influencing meat yield, consumer preference, and production efficiency [3,4]. Understanding the molecular mechanisms that govern the development and function of adipose tissue is therefore fundamental to advancing breeding strategies for optimized carcass composition and improved meat quality.

During postnatal growth, the development of adipose tissue is characterized by dynamic changes in cell proliferation [5], fat deposition and metabolism [6], and extra-cellular matrix remodeling [7]. Despite the physiological importance of this transition, however, the molecular events orchestrating age-dependent changes in backfat development remain largely unknown, and trait-integrated proteomic network analyses that link backfat proteomic profiles to key carcass traits are still lacking in Ningxiang pigs, a typical indigenous fatty breed in China.

Traditional studies on adipose tissue biology have relied heavily on transcriptomic analyses, which offer valuable insights into gene regulatory mechanisms. However, mRNA abundance does not always correlate with protein levels, particularly in complex metabolic tissues where post-transcriptional and post-translational regulation are common. Proteomic analysis, by directly measuring the abundance of functional proteins, provides a more accurate and comprehensive understanding of tissue physiology and metabolic state. Moreover, integrative proteomic approaches that link protein abundance to phenotypic traits can reveal candidate markers and regulatory nodes that drive economically important outcomes such as fat deposition and carcass quality.

The Ningxiang pig, a well-known indigenous Chinese breed, is characterized by its high fat deposition and superior meat quality but relatively slow growth performance [8,9]. This unique phenotype makes it an ideal model for studying the regulation of adipose tissue development in pigs. However, the proteomic basis of fat accumulation and its association with carcass traits in Ningxiang pigs has not been thoroughly explored.

Therefore, the objective of this study was to systematically characterize the proteomic landscape of backfat development in Ningxiang pigs across key postnatal stages using tandem mass tag (TMT)-based quantitative proteomics. We aimed to identify differentially abundant proteins, uncover age-dependent biological pathways, and construct co-abundance networks to reveal key protein modules and hub regulators associated with carcass traits. We hypothesized that specific groups of proteins and hub regulators would show coordinated changes during postnatal growth and exhibit associations with carcass traits, especially lean meat and fat percentage. Our goal was to uncover molecular markers and pathways that could be used to improve genetic selection and management strategies for carcass quality in commercial pig breeding.

## 2. Materials and Methods

### 2.1. Animal Ethics

All animal procedures were conducted in strict accordance with the guidelines approved by the Animal Care and Use Committee of the Hunan Institute of Animal and Veterinary Medicine (Approval No. 20211020). Throughout the experimental period, pigs were maintained under standardized feeding conditions with ad libitum access to feed and water. Animals were cared for by trained husbandry staff under veterinary supervision, following routine immunization and disinfection protocols. Euthanasia was performed humanely via electrical stunning followed by exsanguination.

### 2.2. Animals and Sample Preparation

A total of 18 healthy Ningxiang pigs (barrows) were used, representing six postnatal developmental stages: 60, 120, 180, 240, 300, and 360 days of age (n = 3 per stage). All pigs were weaned and enrolled in the experiment at 30 days of age, then randomly assigned to the designated slaughter age groups. Animals were sourced from Hunan Liushahe Ecological Animal Husbandry Co., Ltd. (Changsha, China), where they were raised under identical management conditions. Pigs were housed in well-ventilated pens with stable environmental control; the temperature was maintained at 20–26 °C and relative humidity at 60–70%. Pigs were provided ad libitum access to feed and water. Dietary composition is shown in Table 1. Average body weights at the respective stages were recorded prior to slaughter.

Backfat tissue was collected within 30 min post-mortem from the thickest point of the shoulder (between the third and fourth ribs). Samples were immediately snap-frozen in liquid nitrogen and stored at −80 °C until proteomic analysis. For proteomic analysis, each pig was treated as an independent biological replicate (n = 3 per age group), and no sample pooling was performed. Thus, each age group consisted of different individuals, and no repeated sampling was performed on the same animal.

### 2.3. Protein Extraction

Backfat tissues were ground in liquid nitrogen and lysed in SDT buffer (4% SDS, 100 mM Tris-HCl, pH 7.6). The lysate was homogenized, sonicated, and boiled for 15 min. After centrifugation at 14,000× *g* for 15 min, the supernatant was filtered through a 0.22 μm membrane. Protein concentration was determined using the BCA protein assay kit (P0012, Beyotime, Shanghai, China), and protein integrity was assessed by sodium dodecyl sulfate-polyacrylamide gel electrophoresis (SDS-PAGE, P0012A, Beyotime, Shanghai, China).

### 2.4. Trypsin Digestion and TMT Labelling

For each sample, 200 μg of protein was diluted with 30 μL SDT buffer. Detergents and low-molecular-weight components were removed using UA buffer (8 M urea, 150 mM Tris-HCl, pH 8.5) via ultrafiltration (30 kDa cutoff, Sartorius, Göttingen, Germany). The concentrate was alkylated with 100 μL iodoacetamide (100 mM in UA buffer) for 30 min in darkness at room temperature. After additional UA and triethylammonium bicarbonate (TEAB) buffer washes, proteins were digested with 4 μg trypsin in 0.1 M TEAB buffer overnight at 37 °C. The resulting peptides were quantified by OD280 using a NanoDrop 3000 (Thermo Fisher Scientific, Waltham, MA, USA). Subsequently, 100 μg peptide per sample was labeled with 41 μL of TMT reagent (Thermo Fisher Scientific, USA) for 1 h at room temperature. The reaction was quenched with 8 μL of 5% hydroxylamine, followed by 15 min incubation. Labeled peptides were pooled for high-pH reversed-phase chromatography. TMT 18-plex reagent was used for labeling, with each of the 18 samples (6 developmental stages × 3 biological replicates) assigned to a unique channel within a single plex. No multiple plexes were applied, so no bridge channel or inter-plex normalization was needed. After pooling, labeled peptides were fractionated by high-pH reversed-phase chromatography.

### 2.5. Peptide Fractionation by High pH Reversed-Phase Chromatography

TMT-labeled peptides were fractionated on a 1260 Infinity II HPLC system (Agilent, Santa Clara, CA, USA) using a high-pH reversed-phase (hpRP) gradient. Samples were diluted in buffer A (10 mM ammonium formate, 5% ACN, pH 10.0) and loaded onto a XBridge Peptide BEH C18 column (130 Å, 5 μm, 4.6 mm × 100 mm). Peptides were eluted at 1 mL/min with a gradient from 0 to 7% buffer B (10 mM ammonium formate, 85% ACN, pH 10.0) over 5 min, 7–40% buffer B from 5 to 40 min, 40–100% buffer B from 40 to 45 min, and held at 100% buffer B for 15 min. Absorbance was monitored at 214 nm, and fractions were collected every minute and dried under vacuum at 45 °C.

### 2.6. LC-MS/MS Analysis

Each fraction was analyzed using an EASY-nLC system (Thermo Fisher Scientific, Waltham, MA, USA) coupled to a Q Exactive Plus mass spectrometer (Thermo Fisher Scientific, Waltham, MA, USA). Peptides were separated on an Acclaim PepMap RSLC C18 column (50 μm × 15 cm, P/N 164943) at a flow rate of 300 nL/min. The gradient consisted of: 6% buffer B for 3 min, 6–28% B over 42 min, 28–38% B over 5 min, 38–100% B over 5 min, and hold at 100% B for 5 min.

Mass spectrometry (MS) analysis was performed on a Q Exactive Plus mass spectrometer (Thermo Fisher Scientific, USA) at LC Sciences (Hangzhou, Zhejiang, China). The mass spectrometer operated in positive ion mode with the following settings: MS1 scan range: 350–1800 *m*/*z*; MS1 resolution: 70,000; AGC target: 3 × 10^6^; Max injection time: 50 ms; MS2 scans: Top 10 data-dependent scans after each MS1; MS2 resolution: 35,000. Isolation window: 2 *m*/*z*; Normalized collision energy: 30 eV; Max MS2 injection time: 45 ms.

### 2.7. Identification of Differentially Abundant Proteins

MS raw data were processed using Proteome Discoverer v2.1 (Thermo Fisher Scientific, Waltham, MA, USA) and searched using the MASCOT v2.6 engine (Matrix Science, London, UK). The MASCOT search parameters were set as follows: precursor mass tolerance ±10 ppm, fragment mass tolerance ±0.05 Da, enzyme specified as trypsin with a maximum of 2 allowed missed cleavages, fixed modification of carbamidomethylation on cysteine residues, and variable modifications of oxidation on methionine residues and TMT labeling on N-termini and lysine residues. Protein identifiers were mapped to gene symbols using the UniProt ID Mapping service. Differentially abundant proteins across developmental stages were identified using one-way ANOVA (*p* < 0.05) with Benjamini–Hochberg FDR correction. Tukey’s HSD post hoc tests were performed for pairwise comparisons. Data visualization was conducted using ggplot2 [10] and pheatmap [11] in R.

### 2.8. Weighted Co-Abundance Network Analysis

We applied a weighted co-abundance network analysis framework, following the core procedures of the WGCNA method [12], to construct protein co-abundance networks. Protein data were normalized prior to analysis. The soft-thresholding power (β = 6) was selected based on scale-free topology (R^2^ = 0.8). The adjacency matrix and topological overlap matrix (TOM) were computed, and hierarchical clustering was used to identify modules. Modules with eigengene correlations > 0.8 were merged. Module-trait relationships were assessed using Pearson correlation with developmental stages and carcass traits from our previously published dataset [13], which was generated from the same cohort of animals used for proteomic profiling in this study. The individual carcass trait values for the 18 animals are provided in Supplementary Table S1. Modules with |R| > 0.7 and *p* < 0.05 were considered biologically significant. Significance of these associations was empirically validated through 1000 permutation tests (*p* < 0.05), confirming the non-random nature of the observed correlations and reinforcing the biological validity of the identified co-abundance modules. Hub proteins were prioritized based on module membership scores. The R code can be accessed from https://doi.org/10.6084/m9.figshare.29597027 (accessed on 18 April 2026).

### 2.9. Gene Ontology (GO) Enrichment and Network Analysis

GO enrichment was conducted using the GO Enrichment Analysis tool (https://geneontology.org/), powered by PANTHER (version 20240807) [14], with the GO Ontology database (release 16 March 2025; DOI: 10.5281/zenodo.15066566). Fisher’s exact test with FDR correction (FDR < 0.05) was applied. Protein–protein interaction (PPI) networks were constructed and visualized using igraph (version 2.0.3) [15] and ggraph (version 2.2.1) [16] packages in R.

### 2.10. Statistical Analysis

Proteomic analysis included three biological replicates per developmental stage. Raw intensity values were log_2_-transformed and normalized via LOESS regression using the limma package (version 3.60.3) [17]. Principal component analysis (PCA) was performed using the prcomp function in R software (version 4.4.2) with log_2_-transformed and LOESS-normalized protein intensity values as input. To statistically validate the grouping patterns observed in PCA, permutational multivariate analysis of variance (PERMANOVA, Adonis test) was conducted using the vegan package (version 2.6-6.1) with 999 permutations. One-way ANOVA with Benjamini–Hochberg FDR correction was used to identify significant changes across stages (*p* < 0.05). Pairwise comparisons were conducted using Tukey’s HSD test. Correlations with traits were considered strong when |R| > 0.7.

## 3. Results

### 3.1. Quality Control and Proteomic Profiling of Backfat Tissue

The quality of the extracted proteins was validated by multiple quality control (QC) assays to ensure reliability of subsequent proteomic analysis. SDS-PAGE analysis confirmed the integrity of protein samples (Figure S1A). The base peak chromatogram exhibited numerous peaks with distinct elution times and high relative abundance (Figure S1B), indicating high complexity of peptides in the samples. Peptide labeling efficiency exceeded 99.4% (Figure S1C), and the mass deviation of more than 99.5% of peptides was distributed within 8 parts per million (ppm) (Figure S1D). Collectively, these QC results demonstrate excellent sample consistency, high peptide labeling quality, and precise mass spectrometry detection—all of which confirm that the data are reliable and suitable for further downstream analysis. All QC plots were generated using the mass spectrometry acquisition and data analysis software (Proteome Discoverer, version 2.5, Thermo Fisher Scientific, Waltham, MA, USA).

**Figure 1:**
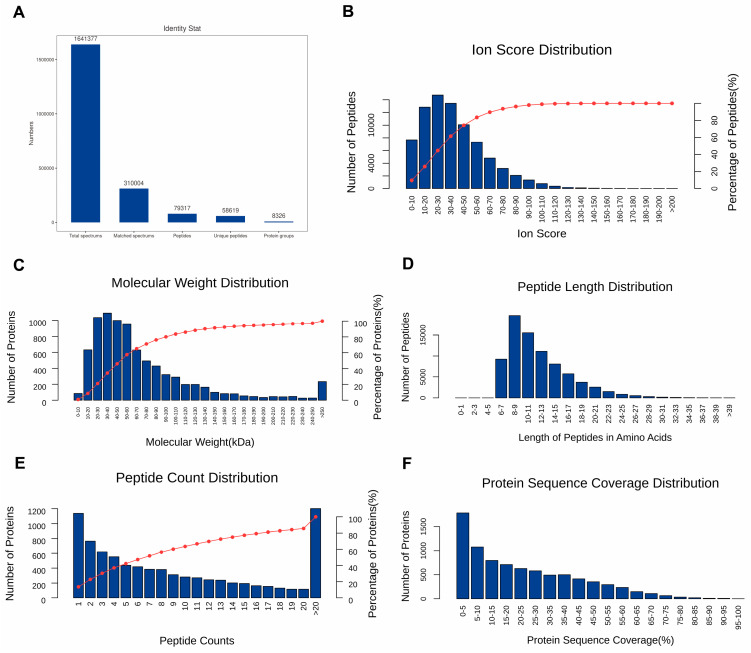
Proteome analysis by using TMT method. (**A**) Identification of spectrums, peptides and proteins. (**B**) Ion score distribution of identified peptides. (**C**) The relative molecular weight distribution of identified protein. (**D**) Length distribution of identified peptides based on amino acids. (**E**) Number distribution of identified peptides. (**F**) Sequence coverage distribution of identified proteins.

The proteome analysis showed 1,641,377 raw spectra, including 310,004 matched spectra, 79,317 peptides, and 8326 proteins (Figure 1A). Moreover, the characteristics of identified peptides and proteins, including ion score of peptides, molecular weight of proteins, length of peptides, count of peptides and sequence coverage of proteins, were comprehensively depicted in Figure 1B–F. Together, these data demonstrate the high quality and reliability of the identified proteome. The lengths of most peptides ranged from 6 to 21 amino acids, with the maximum number being 8–9 amino acids. And more than 80% of the identified proteins contained at least two peptides.

**Figure 2:**
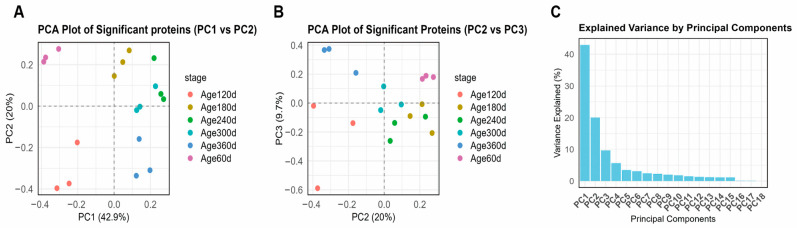
PCA of proteomic profiles across developmental stages (day 60–360). (**A**) PCA Plot of Significant proteins (PC1 vs. PC2). (**B**) PCA Plot of Significant Proteins (PC2 vs. PC3). (**C**) Explained Variance by Principal Components. The first three components explained over 70% of the total variance. Samples clustered by age, with the most pronounced separation between the early developmental stage (day 60–120) and older groups (day 300–360). The 180 d and 240 d stages formed a continuous transitional cluster between them.

Principal component analysis revealed clear age-related separation in protein abundance profiles. The first three principal components (PC1: 42.9%, PC2: 20.0%, PC3: 9.7%) accounted for over 70% of the total variance, with the most pronounced differences observed between the early developmental stage (day 60–120) and older age groups (300–360) (Figure 2). PERMANOVA analysis confirmed that developmental stage significantly influenced the overall proteomic profiles (R^2^ = 0.56, *p* < 0.001), statistically validating the PCA-based clustering patterns. Notably, the 180 d and 240 d stages formed a transitional cluster between the early and older groups rather than separating as distinct extremes. This intermediate positioning aligns with the physiological growth characteristics of Ningxiang pigs: 180–240 days is the critical period when backfat development shifts from hyperplasia to hypertrophy [8,18]. Consequently, the proteomic profile at these stages exhibits a gradual molecular transition from an early growth-dominated pattern to a late fat deposition-dominated pattern, explaining their intermediate position in PCA space.

### 3.2. Functional Enrichment Analysis of Age-Related Proteomic Changes

To further explore the biological processes associated with these age-related changes, we performed GO enrichment analysis on the differentially abundant proteins between the day 60–120 and older age groups. The top 20 GO terms reflected the most relevant biological processes associated with age-related changes in backfat tissue.

**Figure 3:**
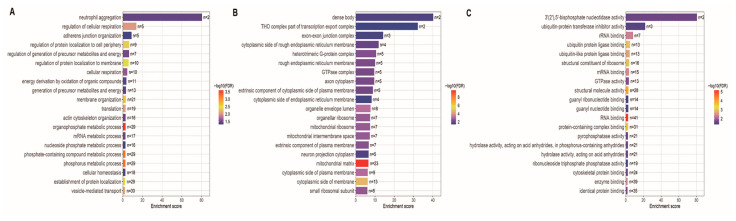
Gene Ontology (GO) enrichment of differentially abundant proteins between day 60–120 and older stages. (**A**) Biological process enrichment revealed significant overrepresentation of energy metabolism, protein localization, and immune-related pathways, including cellular respiration, translation, vesicle-mediated transport, and neutrophil aggregation. (**B**) Cellular component enrichment highlighted mitochondrial- and membrane-associated structures, including mitochondrial ribosomes, endoplasmic reticulum membrane, and ribonucleoprotein complexes. (**C**) Molecular function enrichment identified RNA- and mRNA-binding proteins, structural constituents of ribosomes, and proteins involved in enzyme binding, ubiquitin ligase binding, and GTPase activity.

Biological process enrichment revealed overrepresentation of pathways associated with energy metabolism, protein localization, and immune response (Figure 3A). Specifically, terms such as cellular respiration, translation, vesicle-mediated transport, and membrane organization were significantly enriched, reflecting increased metabolic and biosynthetic activity (Figure 3A). In the context of adipose biology, these changes suggest enhanced mitochondrial oxidative capacity and active lipid droplet dynamics, which are critical for lipid storage and mobilization during backfat maturation. Immune-related processes, including neutrophil aggregation, were also enriched, suggesting heightened immune modulation with age (Figure 3A). Additionally, phosphate- and nucleotide-related metabolic processes were upregulated, indicating enhanced molecular turnover and energy production (Figure 3A).

Cellular component analysis showed enrichment of mitochondrial and membrane-associated structures (Figure 3B). Strong signals were detected for mitochondrial matrix, mitochondrial ribosome, and intermembrane space, pointing to elevated mitochondrial function (Figure 3B). This aligns with the increased metabolic demands of maturing adipocytes, where enhanced mitochondrial activity supports de novo lipogenesis and fatty acid oxidation in backfat tissue. Other enriched components included the rough endoplasmic reticulum membrane, G-protein complexes, and cytoplasmic side of the plasma membrane, highlighting increased activity in protein synthesis, folding, and signal transduction (Figure 3B). Ribosomal complexes and components related to RNA transport, such as the exon-exon junction complex, were also overrepresented (Figure 3B).

Molecular function enrichment further supported these findings, with overrepresentation of RNA binding, mRNA binding, and structural constituent of ribosome, indicating enhanced translation and RNA processing (Figure 3C). Proteins involved in ubiquitin ligase binding and enzyme binding were significantly enriched, suggesting active regulation of protein degradation and cellular signaling (Figure 3C). In adipose tissue, this may reflect the remodeling of the adipocyte proteome during aging, including the turnover of lipogenic enzymes and adipokine secretion machinery. Enrichment of GTPase activity and various hydrolase activities pointed to dynamic energy and phosphate metabolism during this period (Figure 3C).

### 3.3. Identification of Lipid Metabolism Proteins with Age-Dependent Abundance

**Figure 4:**
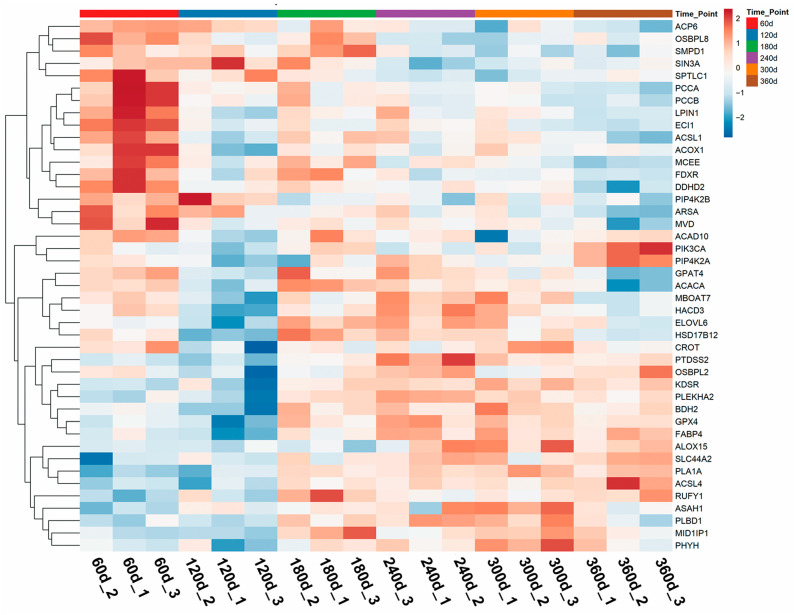
Heatmaps of proteins involved in lipid metabolism across developmental stages. Distinct temporal expression patterns were observed, with the most pronounced transitions occurring between day 60–120 and older age groups.

To delineate the functional impact of age-related proteomic changes on backfat lipid metabolism, we systematically annotated lipid metabolism-associated proteins using the Reactome database. This refined analysis identified 43 key proteins (Table S2) involved in lipid metabolic processes that exhibited significant abundance changes during postnatal development. To visualize the abundance dynamics of these key proteins, heatmaps were generated (Figure 4). The heatmaps revealed distinct abundance patterns across developmental stages, with a marked transition occurring between days 60–120 and older age groups (days 300–360). For enzymes responsible for fatty acid elongation and activation, ELOVL6 abundance rose obviously from days 60–120, peaked at days 180–240, and slowly decreased thereafter until day 360. HACD3 shared a comparable expression trend and reached its maximum level at the middle developmental stage. Differently, ACSL4 was continuously upregulated throughout development and presented the highest abundance at day 360, which implied its central function in sustaining fatty acid activation during intensive lipid deposition. ACSL1 expression fluctuated mildly; it was relatively higher in early stages, decreased at day 120, and remained stable in the subsequent period. Lipid β-oxidation-related proteins exhibited divergent expression patterns. ACAD10 followed a biphasic change: it was highly expressed at day 60, dropped sharply at day 120, recovered at day 180, decreased again at day 300, and finally rebounded at day 360. ACOX1 abundance declined gradually from days 60–240, slightly increased at day 300, and maintained a steady level in the late stage. ECI1 was continuously downregulated with growth, suggesting that the capacity of specific β-oxidation pathways gradually weakened as age increased. As a key lipogenic factor, ACACA experienced a transient upregulation at day 180, which coincided with the accelerated lipid accumulation, followed by a downregulation in later developmental stages.

### 3.4. Co-Abundance Network Analysis Identifies Modules Correlated with Carcass Traits

**Figure 5:**
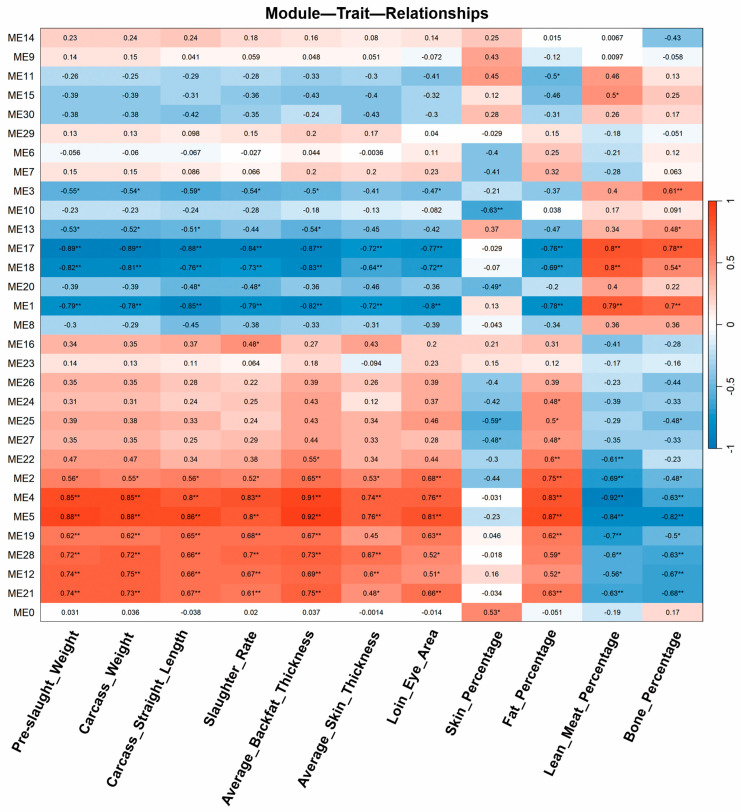
Weighted co-abundance network analysis of the proteomic data. Four significant modules (ME1, ME5, ME17, ME18) were identified with strong correlations to carcass traits. ME5 was positively associated with fat-related traits and negatively correlated with lean meat and bone percentages, while ME1, ME17, and ME18 showed opposite trends. Symbols in the Figure body are explained as follows: * indicates significant correlation (*p* < 0.05), ** indicates highly significant correlation (*p* < 0.01).

To identify protein abundance patterns associated with economically important carcass traits, we performed co-abundance network analysis. This analysis revealed four significant modules—ME1, ME5, ME17, and ME18—that were strongly correlated with carcass composition parameters (n = 18) (Figure 5). Among them, ME5 showed a negative correlation with lean meat percentage and bone percentage, but a positive correlation with fat percentage, carcass weight, and other fat-related traits (Figure 5). In contrast, modules ME1, ME17, and ME18 were positively associated with lean meat and bone percentages, and negatively correlated with fat-related traits (Figure 5).

### 3.5. Temporal Abundance Patterns and Functional Characterization of Module Proteins

**Figure 6:**
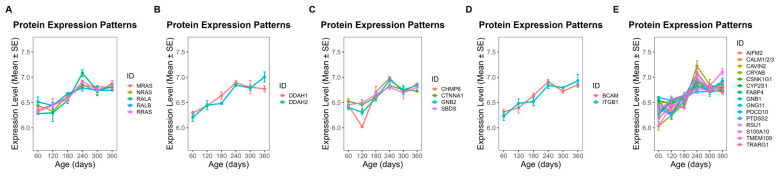
Temporal abundance patterns of proteins in ME5 across developmental stages (day 60–360). Twenty-eight proteins exhibited a steady increase in abundance. GO enrichment revealed significant functional categories: (**A**) GDP binding; (**B**) dimethylargininase activity; (**C**) protein-containing complex binding; (**D**) cell adhesion mediator activity; (**E**) proteins without significant GO enrichment.

**Figure 7:**
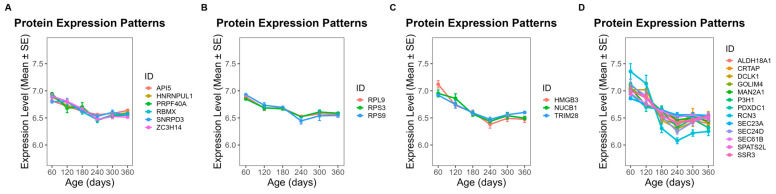
Temporal abundance patterns of proteins in ME1 across developmental stages. Twenty-five proteins displayed a gradual upward trend. GO enrichment revealed significant functional categories: (**A**) RNA binding; (**B**) rRNA binding; (**C**) nucleic acid binding; (**D**) proteins without significant GO enrichment.

**Figure 8:**
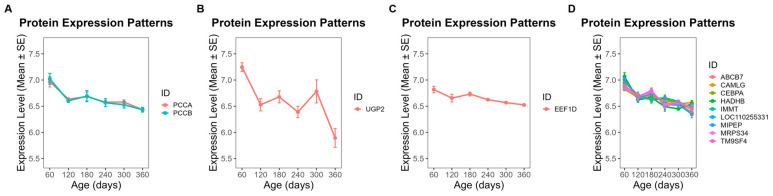
Temporal abundance patterns of proteins in ME18 across developmental stages. Thirteen proteins showed a continuous decline from day 60 to day 360. GO enrichment revealed significant functional categories: (**A**) propionyl-CoA carboxylase activity; (**B**) UTP: glucose-1-phosphate uridylyltransferase activity; (**C**) ligase activity; (**D**) proteins without significant GO enrichment.

**Figure 9:**
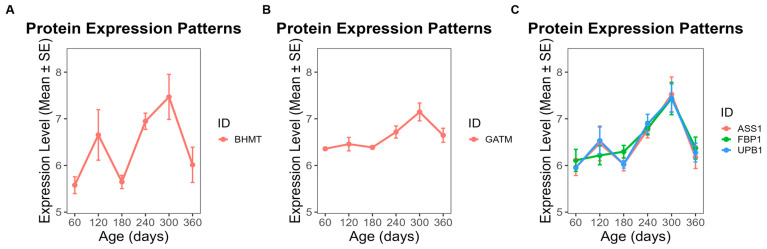
Temporal abundance patterns of proteins in ME17 across developmental stages. Five proteins peaked at day 300 and declined slightly by day 360. GO enrichment revealed significant functional categories: (**A**) betaine-homocysteine S-methyltransferase activity; (**B**) transferase activity related to one-carbon group transfer; (**C**) proteins without significant GO enrichment.

To further investigate the functional implications of these modules, we focused on proteins exhibiting clear temporal abundance patterns across developmental stages (day 60 to day 360). In ME5, 28 proteins showed a steady increase in abundance throughout this period (Figure 6), while 25 proteins in ME1 also displayed a gradual upward trend (Figure 7). By contrast, 13 proteins from ME18 exhibited a continuous decline from day 60 to day 360 (Figure 8). In ME17, five proteins reached peak abundance at day 300 and then declined slightly by day 360 (Figure 9).

GO enrichment analysis was used to classify proteins into functional groups. The enriched terms included: GDP binding, dimethylargininase activity, protein-containing complex binding, and cell adhesion mediator activity for ME5 (Figure 6); RNA binding, rRNA binding, and nucleic acid binding for ME1 (Figure 7); propionyl-CoA carboxylase activity, UTP:glucose-1-phosphate uridylyltransferase activity, and ligase activity for ME18 (Figure 8); and betaine-homocysteine S-methyltransferase activity and transferase activity (one-carbon group transfer) for ME17 (Figure 9). Proteins without significant enrichment were grouped separately in each figure.

### 3.6. Identification of Hub Proteins Within Co-Abundance Modules

**Figure 10:**
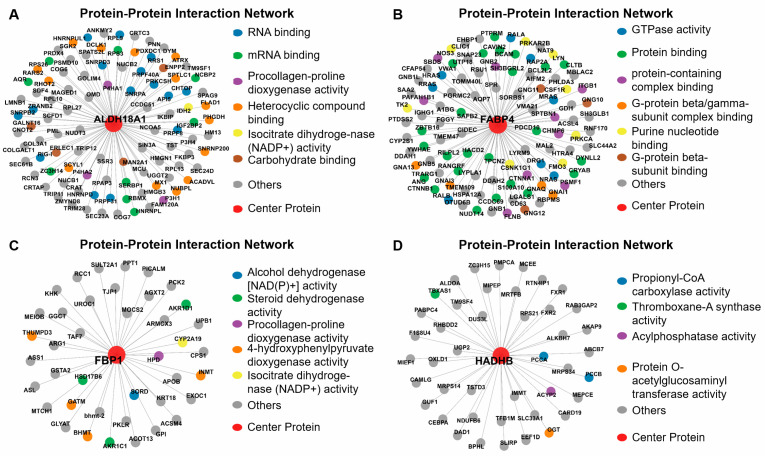
Identification of hub proteins within key co-abundance modules. (**A**) ME1: ALDH18A1; (**B**) ME5: FABP4; (**C**) ME17: FBP1; (**D**) ME18: HADHB. Intramodular connectivity analysis identified these four hub proteins as putative regulators of age-related adipose proteomic changes.

Through intramodular connectivity analysis within the co-abundance network framework, we identified four key hub proteins—aldehyde dehydrogenase 18 family member A1 (ALDH18A1), fatty acid binding protein 4 (FABP4), Fructose bisphosphatase 1 (FBP1), and hydroxyacyl-CoA dehydrogenase trifunctional multienzyme complex subunit beta (HADHB)—each representing central nodes within the ME1, ME5, ME17, and ME18 modules, respectively (Figure 10).

## 4. Discussion

This study provides a comprehensive proteomic analysis of age-related changes in backfat tissue of Ningxiang pigs from day 60 to day 360. Principal component analysis and differential abundance analysis revealed clear separation between early (days 60–120) and later age groups. GO enrichment analysis showed that these proteins were predominantly involved in energy metabolism, protein synthesis, immune response, and membrane organization. Cellular component analysis highlighted enrichment in mitochondrial structures and ribosomal complexes, while molecular function terms included RNA binding, GTPase activity, and ubiquitin-related functions. In addition, 43 proteins significantly enriched in lipid metabolism were identified. Co-abundance network analysis further defined four key protein modules (ME1, ME5, ME17, ME18) that were significantly correlated with lean meat percentage, fat percentage, carcass weight, etc. Finally, four hub proteins, FABP4, FBP1, ALDH18A1, and HADHB, were identified as central nodes within these key modules.

### 4.1. Proteomic Profiling Reveals a Developmental Shift in Backfat Tissue from Early Growth to Late Lipid Deposition

The identification of several lipid metabolism–related proteins provide further insight into how adipose tissue function evolves during postnatal development. The pronounced shift in their abundance between early and late stages supports a transition from a growth-dominated state to one characterized by lipid storage. This pattern is consistent with classical observations that early adipose development is driven primarily by adipocyte hyperplasia, whereas later stages are dominated by hypertrophy and lipid accumulation [19].

At the molecular level, increased abundance of key lipogenic enzymes such as ACACA indicates enhanced capacity for de novo fatty acid synthesis at later stages. As rate-limiting enzymes in lipogenesis, their upregulation is widely regarded as a hallmark of active lipid deposition [20]. In parallel, changes in enzymes involved in fatty acid activation and elongation, including ACSL1/4, ELOVL6, and HACD3, point to ongoing remodeling of fatty acid composition, which is essential for maintaining membrane properties and supporting triglyceride storage [21,22]. In particular, ELOVL6 has been implicated in regulating fatty acid chain length and systemic metabolic homeostasis [23]. Despite this shift toward lipid accumulation, proteins associated with β-oxidation (e.g., ACOX1, ECI1, and ACAD10) remained differentially expressed, indicating that lipid catabolism is maintained alongside synthesis [24,25,26]. This coexistence of anabolic and catabolic processes suggests a dynamic metabolic balance, a characteristic feature of maturing adipocytes [27].

The intermediate positioning of the days 180–240 in PCA further supports a gradual transition in adipose tissue function. This suggests that proteomic remodeling reflects a progressive reprogramming of metabolic priorities, requiring coordinated regulation of lipid synthesis, oxidation, and mitochondrial activity to meet changing energy demands. Beyond metabolism, the enrichment of mitochondrial, translational, and immune-related processes indicates broader cellular remodeling. Increased mitochondrial activity is closely linked to both adipogenesis and lipid turnover, providing the necessary energy and substrates for biosynthesis [27]. At the same time, immune-related signatures are consistent with evidence that adipose tissue expansion is accompanied by immune cell recruitment and low-grade inflammation, even under physiological conditions [28,29]. The concurrent upregulation of RNA processing and protein turnover pathways further suggests enhanced proteome plasticity, enabling adipocytes to adapt to developmental and metabolic cues. Together, these findings indicate that backfat maturation is an integrated process involving metabolic reprogramming, immune modulation, and continuous cellular adaptation.

### 4.2. Proteomic Network Analysis Highlights Key Candidate Regulators of Energy Homeostasis Linked to Carcass Traits

To link proteomic changes with economically important phenotypes, we applied co-abundance network analysis and identified four key co-abundance modules (ME1, ME5, ME17, ME18) that were significantly correlated with carcass traits. Time-course analysis of module-specific proteins revealed coherent abundance patterns that further support their biological relevance. For example, the abundance of ME5 and ME1 proteins predominantly increased with age, suggesting roles in adipose tissue expansion and muscle development, respectively. Conversely, the abundance of several ME18 proteins declined over time, possibly reflecting their involvement in early growth processes that taper off with maturation. Additionally, four hub proteins, FABP4, FBP1, ALDH18A1, and HADHB, were identified in these 4 co-abundance modules.

Within module ME1, ALDH18A1 emerged as a central hub protein with strong connectivity to several proteins involved in extracellular matrix (ECM) organization and collagen biosynthesis. As a key enzyme in the proline biosynthetic pathway, ALDH18A1 catalyzes the conversion of glutamate to pyrroline-5-carboxylate, an amino acid critical for collagen synthesis [30]. ALDH18A1 exhibited network correlation with prolyl 3-hydroxylase 1 (P3H1) and cartilage associated protein (CRTAP), both of which are integral to post-translational collagen modification [31]. Additionally, collagen beta(1-o)galactosyltransferase 1 (COLGALT1), a collagen-specific galactosyltransferase, contributes to the maturation of collagen triple helices through glycosylation [32], further underscoring the ECM-focused role of this protein network. The association of SEC23A, a component of the COPII complex responsible for vesicular transport from the endoplasmic reticulum [33], points to an active secretion pathway for collagen and ECM proteins. This is consistent with previous transcriptomic studies in Ningxiang pigs, which identified multiple ECM-related hub genes (e.g., *FN1*, *DCN*, *COL1A1*, *COL5A1*) during backfat development [18].

FABP4, the hub protein of ME5 module, is a well-known adipocyte-specific protein involved in fatty acid intracellular transport and metabolism [34]. The G-protein signaling components, GNB2, GNAI1 and GNAQ, showed high correlation with FABP4. These proteins are central to G-protein–coupled receptor signaling, which mediates lipolysis and insulin sensitivity in adipose tissue [35]. Their co-abundance with FABP4 suggests tight coordination between lipid handling and hormonal signal transduction in metabolically active adipocytes. Alpha-B-crystallin (CRYAB), a small heat shock protein, is also correlated with FABP4. CRYAB is involved in protecting adipocytes from oxidative stress and may support lipid droplet stability during adipocyte hypertrophy [36]. This aligns with the role of FABP4 in regulating intracellular fatty acids and mitigating lipotoxicity. Notably, FABP4 has been reported to be more highly expressed in fatty indigenous pig breeds than in lean commercial breeds, confirming its key role in fat deposition [37]

FBP1, the hub protein of ME17 module, is a key enzyme in gluconeogenesis, catalyzing the hydrolysis of fructose-1,6-bisphosphate to fructose 6-phosphate [38]. While traditionally considered a hepatic enzyme, increasing evidence supports its presence in adipose tissues, where it plays roles in carbohydrate flux and energy balance in adipocytes [38]. In this study, FBP1 showed associations with several metabolic enzymes, such as pyruvate kinase L/R (PKLR) and phosphoenolpyruvate carboxykinase 2 (PCK2), which are also involved in glycolysis and gluconeogenesis [39,40], reinforcing its metabolic regulatory function. FBP1 was also connected with aldo-keto reductase family 1 member D1 (AKR1D1) and glutathione S-transferase alpha 2 (GSTA2), enzymes in-volved in steroid metabolism [41] and detoxification pathways [42], respectively. The connectivity profile of FBP1 underscores a coordinated glucose-lipid interplay in expanding adipose tissue, potentially enabling adipocytes to balance energy storage with glucose availability and oxidative demands. In line with prior transcriptomic analyses of Ningxiang pigs, FBP1 is involved in glucose-lipid crosstalk during backfat development [18].

HADHB, the hub protein in ME18 module, is a mitochondrial enzyme essential for mitochondrial energy production through fatty acid degradation [43]. In adipose tissue, HADHB expression indicates enhanced fatty acid oxidation capacity and energy mobilization [44]. The observed decline in HADHB protein levels from day 60 to 360 in this study is therefore consistent with a developmental shift in metabolic priority. Specifically, it may reflect a transition from an early postnatal state characterized by active lipid catabolism and energy expenditure towards a later state dominated by energy storage and lipid accumulation in mature adipose tissue. The negative association between HADHB level and fat-related traits further supports its role as a marker of energy mobilization, with lower abundance facilitating a pro-storage phenotype. This pattern aligns with the understanding that fatty pig breeds exhibit lower fatty acid oxidation capacity compared to lean breeds during later developmental stages [18].

HADHB network is co-expressed with many mitochondrial proteins in this study. For instance, it is correlated with ATP binding cassette subfamily B member 7 (ABCB7), an iron transporter regulates mitochondrial iron homeostasis [45]. It was also associated with propionyl-CoA carboxylase subunit alpha and beta (PCCA and PCCB), which regulate branched-chain amino acid and odd-chain fatty acid catabolism [46]. Notably, mitochondrial intermediate peptidase (MIPEP), Mitochondrial Ribosomal Protein S34 and S14 (MRPS34 and MRPS14) were found within HADHB network, which involves in regulating mitochondrial protein translation and maturation [47,48]. HADHB also displayed associations with AlkB Homolog 7 (ALKBH7), a mitochondrial α-ketoglutarate-dependent dioxygenase implicated in apoptosis and lipid metabolism [49,50]. Together, these network associations highlight HADHB as a central candidate regulator of mitochondrial energy metabolism in adipose tissue, coordinating fatty acid oxidation with broader mitochondrial functions such as protein maturation, iron homeostasis, and apoptotic signaling.

The four hub proteins represent distinct yet interconnected biological functions that contribute to lipid metabolism and energy homeostasis in backfat tissue. FABP4 and FBP1 are primarily associated with lipid storage and glucose metabolism, respectively, while ALDH18A1 and HADHB reflect structural and catabolic processes that support adipose tissue expansion and remodeling. Their co-abundance with key proteins involved in extracellular matrix organization, mitochondrial function, and hormonal signaling suggests coordinated regulation of metabolic and developmental programs in adipose tissue. The significant correlations between these hub proteins and carcass traits highlight their potential not only as functional candidate regulators but also as potential biomarkers or targets for genetic selection. These findings help explain how changes in protein abundance are connected to important traits like fat levels and growth. It is important to acknowledge the breed-specific context of these findings. As a valuable indigenous breed with inherent high fat deposition, Ningxiang pigs exhibit distinct phenotypic traits compared to modern lean-type commercial breeds. Consequently, the hub proteins (ALDH18A1, FABP4, FBP1, and HADHB) and their associated co-abundance networks identified here are most directly relevant to indigenous breeds with similar fat metabolism or to crossbreeding programs involving indigenous ancestry.

Collectively, these observations align well with conserved molecular signatures that distinguish fatty indigenous pigs from lean commercial breeds [51,52]. Compared with lean breeds such as Landrace, fatty Chinese indigenous pigs consistently show higher abundance of proteins promoting lipid uptake and storage (FABP4, FBP1) and lower abundance of proteins driving fatty acid oxidation (HADHB). Such a pattern reinforces enhanced fat deposition and reduced lipid catabolism as core characteristics of the fatty phenotype [51,52]. As a typical Chinese fatty indigenous breed, Ningxiang pigs follow this conserved regulatory logic, and the hub proteins identified herein may serve as shared molecular markers for fat deposition traits in Chinese indigenous pig breeds.

## 5. Conclusions

In summary, this study delineates the age-dependent remodeling of the backfat proteome in Ningxiang pigs. Our analyses reveal a distinct developmental shift between early and late growth stages and identify key pathways, co-abundance modules, and hub proteins associated with fat deposition and energy homeostasis. The resulting proteomic dataset and candidate list establish a valuable resource and a framework for future mechanistic studies and breeding strategies aimed at improving carcass quality.

## Figures and Tables

**Table 1:** Diet composition and nutrition level.

Items	Contents (%)
Ingredient	
Corn	64.00
Soybean meal (CP43)	16.00
Wheat bran	16.00
Premix ^①^	4.00
Nutrition level ^②^	
Digestive energy (MJ/kg)	15.20
Crude protein	14.00
Calcium	14.00
Total phosphorus	0.55

Note: ^①^ Premix provided per kg of diet: Fe 110.00 mg, Cu 10.00 mg, Se 0.32 mg, Zn 110.00 mg, Mn 10.00 mg, vitamin A 930.80 μg, vitamin D_3_ 8.50 μg, vitamin E 14.20 mg, vitamin B_2_ 4.00 mg, niacin 23.00 mg, pantothenic acid 15.00 mg, vitamin B_12_ 0.04 mg, biotin 0.10 mg, choline 120.00 mg. ^②^ Nutrient levels were calculated values.

## Data Availability

The mass spectrometry proteomics data generated in this study have been deposited to the ProteomeXchange Consortium via the PRIDE partner repository with the dataset identifier PXD067020. The data are privately accessible for review purposes using Project Accession PXD067020 and Reviewer Token OsAWDiMce6dC. The supporting R code is privately hosted on figshare and is available for review via the following link: https://doi.org/10.6084/m9.figshare.29597027 (accessed on 18 April 2026). Both datasets will be made publicly available upon acceptance of the manuscript.

## Supplementary Materials

The following supporting information can be downloaded at: https://www.mdpi.com/article/10.3390/ani16091309/s1, Figure S1: Quality analysis of the extracted proteins. (A) SDS-PAGE of protein in each sample. (B) The labelling efficiency of peptides. (C) TMT-labeling liquid chromatography with tandem mass spectrometry (LC-MS-MS) base peak chromatogram. (D) Quality deviation of peptides; Table S1. Individual carcass trait values of the 18 animals included in the WGCNA; Table S2: List of 43 key lipid metabolism-related proteins identified in Ningxiang pig backfat.
