# Supplementary material for: Age-Driven Proteomic Networks in Ningxiang Pig Backfat Identify Candidate Regulators of Carcass Traits
Source: Animals (Basel). 2026 Apr 24;16(9):1309. doi: 10.3390/ani16091309 (PMC13162576; doi:10.3390/ani16091309)
Supplement: Supplementary file 1 [file animals-16-01309-s001.zip › animals-4234054-supplementary.pdf]

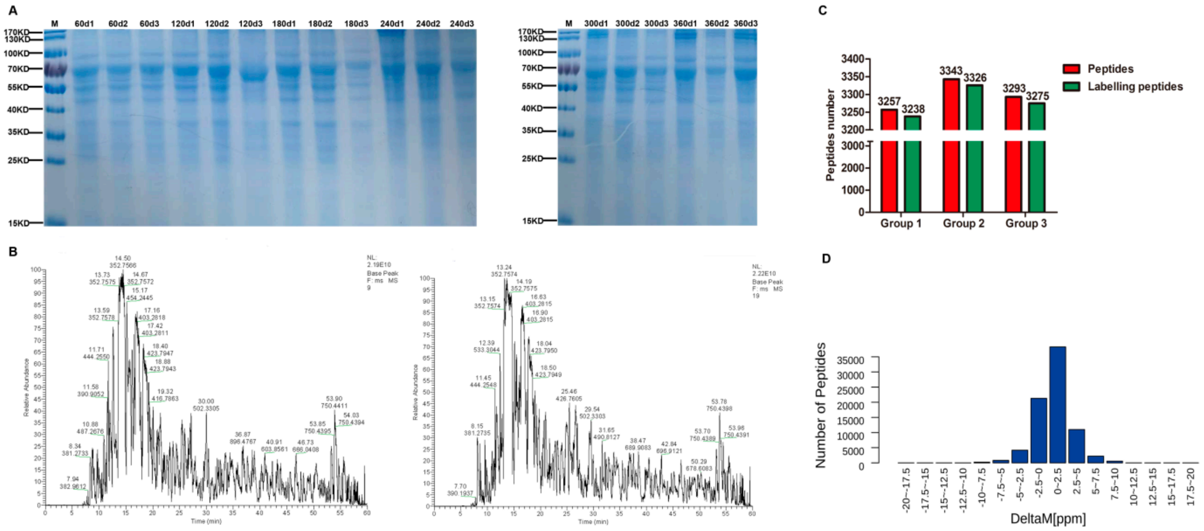

Figure S1. Quality analysis of the extracted proteins. (A) SDS-PAGE of protein in each sample. (B) The labelling efficiency of peptides. (C) TMT-labeling liquid chromatography with tandem mass spectrometry (LC-MS-MS) base peak chromatogram. (D) Quality deviation of peptides.

Table S1. Individual carcass trait values of the 18 animals included in the WGCNA.

Sample ID	Pre-slaught_Weight	Carcass_Weight	Carcass_Straight_Length	Slaughter_Rate	Average_Backfat_Thickness	Average_Skin_Thickness	Loin_Eye_Area	Skin_Percentage	Fat_Percentage	Lean_Meat_Percentage	Bone_Percentage
60d_1	16.4	11	48.5	67.07	16.37	2.38	5.81	11.36	29.24	48.23	11.17
60d_2	17.3	11.32	49.5	65.43	14.63	2.63	7.29	11.75	24.81	51.31	12.13
60d_3	17.35	11.56	49.4	66.63	15.37	2.8	8.35	16.51	20.55	50.46	12.48
120d_1	34.7	23.55	63	67.87	22.23	2.99	12.56	15.88	28.99	44.97	10.16
120d_2	31.95	22.15	61	69.33	20.52	3.06	12.32	14.08	26.81	49.47	9.64
120d_3	36.15	25.1	66.5	69.43	21.04	3.46	10.76	13.55	26.76	49.87	9.82
180d_1	50.1	33.6	70.5	67.07	31	3.34	14.38	8.72	38.18	45.13	7.96
180d_2	55.9	38.95	78	69.68	29.29	4.46	18.74	12.66	29.26	47.87	10.21
180d_3	53	37.1	69.5	70	35.55	3.42	16.75	10.47	38.45	43.23	7.85
240d_1	74.35	53.6	74	72.09	52.16	4.72	14.41	13.4	40.37	37.26	8.96
240d_2	74	54.44	83.5	73.57	39.36	4.25	19.76	11.27	42.36	36.96	9.41
240d_3	72.8	53.26	77	73.16	42.9	4.37	17.57	12.08	44.71	34.75	8.47
300d_1	93.2	67.6	87	72.53	41.37	7.27	17.2	13.32	38.34	38.9	9.44
300d_2	96.05	72.9	90.5	75.9	40.6	6.43	14.78	15.41	35.66	40.47	8.45
300d_3	92.3	69.95	82	75.79	52.37	7.03	18.28	11.58	39.68	41.41	7.33
360d_1	111.5	82.8	91.5	74.26	47.56	5.19	22.01	11.31	40.53	40.48	7.68
360d_2	108.5	81.1	92	74.75	51.03	5.79	16.61	16.02	36.56	39.49	7.93
360d_3	106.9	79.2	88	74.09	52.5	5.69	18.26	12.6	41.59	38.1	7.71

Table S2. List of 43 key lipid metabolism-related proteins identified in Ningxiang pig backfat.

Protein names				
PIP4K2B	FDXR	PCCB	OSBPL8	ASAH1
ECI1	PHYH	MCEE	MID1IP1	PIP4K2A
MBOAT7	FABP4	DDHD2	PLA1A	PLBD1
ACSL1	KDSR	HSD17B12	ACAD10	PLEKHA2
ACACA	CROT	SPTLC1	PIK3CA	PCCA
ALOX15	SIN3A	GPAT4	GPX4	SLC44A2
ACSL4	OSBPL2	RUFY1	HACD3	ELOVL6
BDH2	ARSA	LPIN1	MVD	
ACP6	ACOX1	SMPD1	PTDSS2	
